# Determining Optimal Routes to Surgery for Borderline Resectable Venous Pancreatic Cancer—Where Is the Least Harm and Most Benefit?

**DOI:** 10.3389/fonc.2019.01060

**Published:** 2019-10-17

**Authors:** Rupaly Pandé, Keith J. Roberts

**Affiliations:** ^1^ Department of HPB Surgery and Liver Transplant, University Hospitals of Birmingham NHS Foundation Trust, Birmingham, United Kingdom; ^2^Department of Immunology and Immunotherapy, University of Birmingham, Birmingham, United Kingdom

**Keywords:** borderline resectable pancreaic adenocarcinoma, vein resection and reconstruction, neoadjuvant and adjuvant chemotherapies, upfront surgery, fast track surgery

## Abstract

Surgery among patients with borderline resectable pancreatic cancer (BRPC) and venous disease has emerged as a viable strategy to achieve curative treatment. By definition, these patients are at increased risk of a positive resection margin, however, controversy exists with regards to necessity of radical surgery and optimum pathways with no consensus on definitive treatment. A surgery first approach is possible though outcomes vary but patients can have an efficient pathway to surgery, particularly if biliary drainage is avoided which limits overall complications. Neoadjuvant therapy (NAT) is emerging as a widely used strategy to improve oncological outcomes, including resection margin status. However, some patients progress on NAT whilst others suffer major complications whilst elderly patients are unlikely to be offered effective NAT limiting the widespread applicability of this therapy. In this article an overview of the entire pathway is presented along with assimilation of current best evidence to determine optimal routes to surgery for BRPC with venous involvement.

## Introduction

*Primum non nocere - first do no harm*. Attempts to achieve cure among patients with borderline resectable pancreatic cancer (BRPC) are certainly associated with harm (1–3). Neoadjuvant therapy (NAT) for BRPC offers a new treatment direction and hope for patients with this disease. It is however essential to consider the entire patient pathway to determine where the current balance of benefit lies as this may not be immediately apparent.

Outcomes of established treatment (surgery then adjuvant chemotherapy) can be viewed as marginal (2, 4) with disappointing disease free interval and median overall survival (3, 5) and as such there is a body of thinking that surgery and adjuvant chemotherapy representing the current gold standard treatment of BRPC (6), is essentially flawed. Low rates of achieving an R0 resection (4, 7) or failure to tackle potential micro-metastatic disease (8) are cited as evidence for this. Indeed, MRI (9) or PET (10) imaging are recommended to identify metastatic disease at presentation (6). Furthermore, a matter of weeks delay to treatment is associated with an increased risk of cancer progression (11–14) whilst around 25–50% of patients fail to receive adjuvant therapy following surgery (15–19).

It is easy, therefore, to see the argument against a surgery first approach for BRPC. The alternative, NAT, aims to treat micro-metastatic disease and control cancer at the surgical margin, which by definition in BRPC involves key vascular structure(s) and essentially avoids surgery for patients harboring undetected distant disease or rapidly aggressive disease who would succumb to early recurrence. This strategy is not without precedent as NAT is the gold standard approach to treating esophageal adenocarcinoma and rectal cancer among others (20–23).

Indeed, there are many reports of NAT in pancreatic cancer showing remarkable survival data when compared with patients undergoing a surgery first approach (overall survival 26.1–37.7 vs. 15.0–25.1 months) (24, 25). However, over a third of patients incur grade 3 or greater toxicity following NAT and may not complete treatment whilst other patients suffer disease progression whilst on therapy (26); unless an intention to treat analysis is presented, survival data among patients undergoing surgery after NAT reflects a well-selected cohort and is misleading.

There are therefore fundamental issues to be considered and it is essential to critically appraise existing pathways, including the need to treat jaundice and reported outcomes to decide where the balance of risk and benefit lies in treating patients with BRPC.

## Defining BRPC and Approaches to Surgical Management

A basic requirement of cancer surgery is to remove all cancer without evidence of microscopic disease at the surgical margin. For BRPC it is clear that by definition a standard pancreatoduodenectomy will not achieve this aim (Figure S1). Cancer recurrence is related to margin involvement in pancreatic cancer surgery (15, 27–31). There are several systems proposed to define BRPC, all of which describe the relationship between the tumor and local vascular structures (32–36). A potential failing of existing systems is not to classify venous and arterial involvement as different entities. The ISGPS, however, recognizes that treatment of BRPC can be different depending on whether there is venous or arterial involvement and recommends a surgery first approach for BR venous but not arterial disease (32).

Considering upfront surgery for BRPC and venous resection, some studies have reported increased morbidity (37) and no survival benefit (38), inviting interest for exploring NAT. Following NAT, it is not known whether patients are more or less likely to undergo venous resection, though the operation may be more technically challenging, particularly after radiotherapy, and patients have a longer hospital stay compared to those without venous resection (39, 40). Other groups, however, have shown upfront venous resection of BRPC to be safe and to achieve the same survival rates as patients undergoing upfront surgery for resectable PDAC (41). Conceptually, therefore, if a surgeon can perform venous resection and reconstruction there is no major barrier to performing upfront surgery for those with BRPC and venous disease. A multicenter study and meta-analysis (41, 42) have examined synchronous venous resection, compared to standard pancreatoduodenectomy for PDAC, and demonstrated no difference in morbidity or mortality as well as equivalent survival at 1 and 3 years, supporting this approach and the position of the ISGPS.

However, the approach to BRPC with arterial disease and upfront resection has failed to achieve acceptance as a standard approach due to prohibitive levels of morbidity and mortality (43, 44) with associated arterial resection. Furthermore, among survivors there does not appear to be an equivalent survival advantage to those with “resectable” disease (45, 46).

Accurate disease staging is therefore clearly key to determining the extent of local and metastatic disease. In this regard endoscopic ultrasound can be useful in determining arterial involvement when cross sectional radiology is equivocal (47). There is increasing evidence that a subset of patients have occult metastatic disease at presentation; PET-CT (10) and MRI (48) of the liver have been demonstrated to up-stage patients. Whether all patients should be screened in this manner or whether a selected group should be is unclear; the argument against this is to avoid treatment delays. Assessment with CA19-9 levels can be useful (49) though this is most effective when assessing the efficacy of NAT (50). It is clear that the radiologic response of NAT to chemotherapy is often unreliable (see below) whilst the response to CA19-9 offers a way to assess the biological response of the tumor to therapy.

Whilst surgeons must be able to offer surgery with a high likelihood of achieving R0 margins it is clear that radiologic response to NAT, using the Response Evaluation Criteria in Solid Tumor (RECIST) (51) is not reliable. The radiological and pathological response to treatment may not be aligned due to edema and inflammation of tissues and as such RECIST may over estimate tumor burden (52). In a retrospective study by Katz et al. of 122 patients who underwent NAT, only one patient demonstrated an objective response to NAT yet 85 patients who underwent resection had a 95% R0 resection margin rate (53). A meta-analysis of patients undergoing studies in neoadjuvant chemotherapy with or without radiotherapy for BRPC (26) demonstrated that radiologic evidence of downstaging was uncommon. However, in those undergoing resection (55%) the R0 resection rate was 80% demonstrating the failure of radiology alone to determine response to treatment.

## Survival, Treatment and Selection Bias

Survival following surgery with adjuvant chemotherapy is well-described with modest improvements with the evolution of gemcitabine based regimens (2, 3, 54). Furthermore, it is clear that from population based studies many patients fail to receive these agents (16, 17, 55, 56). The experience of patients receiving NAT and surgery, however, provide a remarkable contrast and eye-catching duration of survival are reported following NAT and surgery. Most series report median survival for operated patients in excess of 30 months, beyond that of patients with resectable disease receiving adjuvant therapy (3). NAT is associated with an increase in the likelihood of achieving an R0 resection supporting this observation. In a meta-analysis of neoadjuvant vs. upfront surgery among patients with BRPC there was a R0 rate of 88.6 and 63.9 in the neoadjuvant and resected groups, respectively (24). However, if one includes all patients undergoing treatment, from an ITT perspective, the proportion of patients achieving an R0 resection the rate falls as not all patients who begin a treatment pathway ultimately undergo surgery. In the Versteijne meta-analysis this was from 88.6 to 57.6% among BRPC cohort (as the resection rate was 65.0%) and from 65.0 to 54.5% among those with upfront surgery for BRPC (as the resection rate was 85.3%). It could be argued that the R0 rate associated with upfront surgery in the Versteijne study may under report margin involvement compared to studies reporting a thorough pathologic assessment of resection margins (57); however, the resection rate in the NAT group is high at 65% with many other series reporting much lower rates of surgery (26, 58). Thus, based on margin involvement alone, there does not presently appear to be an overall advantage between NAT or upfront surgery when all treated patients are considered.

The failure of classification systems to distinguish BR-venous from BR-arterial involvement is a clear weakness of the literature. It is almost impossible to see how an upfront surgical approach can be associated with high rates of R0 margins among patients with BR-arterial disease, whereas shown above, en-bloc resection of BR-venous disease is feasible and can be reproduced between centers (41). Miura et al. has shown that although BRPC is an independent indicator of poor prognosis, once resected there was no difference in overall survival on an intention to treat analysis suggesting that preoperative staging does not influence outcome (59).

Survival must also be considered on an intention to treat basis. There is a much greater likelihood of not undergoing resection within a NAT pathway than when patients undergo upfront surgery (24, 60, 61). Disease progression can affect patients in either pathway but the duration between presentation and surgery is much longer among those receiving NAT. It is clear that despite the apparent advantages of FOLFIRINOX based regimens many patients still progress whilst undergoing therapy (62). Furthermore, FOLFIRINOX based regimens result in significant rates of toxicity. Thus, patients may fail to undergo surgery either due to disease progression associated with a failure of therapy or directly due to harm and frailty, as a consequence of toxicity (63). A review by Winner et al. (64) highlighted the inconsistency of reporting successful delivery of treatment regimens whereby some studies reported <50% of doses being given as well as regimen modifications, delays due to toxicity or intercurrent morbidity causing further delays. Some 27% of patients progressed on therapy with a further 14% were not reaching surgery due to toxicity and/or patient refusal. Versteijne et al. (24) also highlighted that adjuvant therapy was initiated in 68.6% of patients who received upfront surgery compared to only 31% in the NAT group again highlighting the physiological impact of NAT on patients.

A meta-analysis of trials among patients with BRPC (24) demonstrate a superior survival advantage of NAT among patients with BRPC (median overall survival 19.2 vs. 12.8 months, NAT vs. upfront surgery, respectively) but not resectable cancer (17.4 vs. 18.2 months). The combination of BR-venous and BR-arterial disease within the vast majority of studies, however, makes it impossible to determine whether there is a difference of experience and survival between patients with BR-venous and BR-arterial disease in that study. Hirono et al. (65) reported a higher resection rate and median overall survival among patients with BR-venous disease undergoing upfront surgery when compared to those with BR-arterial disease suggesting that there is.

Finally, it is also essential to consider the impact of treatment and selection bias. FOLFIRINOX has established itself as the standard regime for NAT (with or without radiotherapy) (24). However, given that pancreatic cancer is mainly a disease of the elderly, current clinical trials have underrepresented this important demographic. Their comorbidities and performance status would render the toxicity profile of FOLIRINOX more potent (66) and therefore unacceptable hence these studies may not be applicable to the majority of patients (67). Thus, selection bias means that cohorts of patients undergoing NAT tend to be younger and fitter than an unselected cohort of patients with BRPC. In an attempt to overcome this potential selection bias a propensity matched analysis among patients with *resectable* PDAC was conducted by Mokdad et al. (68). That study adjusted for key demographic and cancer stage variables and concluded that survival with NAT was superior to upfront surgery (26 vs. 21 months). However, it failed to match the nature of chemotherapy within the propensity matched model; there was a significant disparity, some 47% of patients received multiagent therapy in the NAT cohort compared to 19.4% in the adjuvant cohort. Furthermore, when patients who received no adjuvant therapy (33% of the upfront surgery cohort) were excluded from the analysis there was no significant difference in survival; that study was also criticized as it failed to include patients who began NAT but did not undergo surgery leading to clear problems with immortal time bias (69).

There is also treatment bias within the studies comparing outcomes among patients receiving NAT or upfront surgery. Most patients receiving NAT have been treated with FOLFIRINOX based regimens and the outcomes of these patients are being compared with patients who have received adjuvant therapy largely based upon gemcitabine (19, 66, 70–72). The efficacy of FOLFIRINOX as an adjuvant agent has only recently been reported (73); demonstrating a large jump in survival compared with gemcitabine based regimens. There is thus treatment bias within the majority of studies comparing NAT with upfront surgery and adjuvant therapy. It appears likely to be less important to consider the timing of chemotherapy and more important to focus upon delivering the most effective forms of therapy.

## “Fast Track” Surgery and PBD

There is a further fundamental aspect of care which affects patient outcome and experience. Most patients with BRPC have jaundice at presentation. Typically, jaundice is treated by preoperative biliary drainage (PBD). There is no similar requirement to treat altered physiology among patients who receive NAT as a standard of care such as esophageal or colorectal cancer. This adds complexity for any patient with PDAC who receives NAT—they must undergo biliary drainage first. This takes time to organize, is unpleasant and potentially harmful (74). Randomized trials clearly demonstrate that harm is associated with PBD. This harm is not trivial. Many patients require readmission, further procedures and some suffer significant complications such as pancreatitis, cholangitis and death (74, 75). Proceeding directly to surgery enables removal of the primary tumor, avoidance of biliary drainage, and its complications; by its nature this can only be offered with upfront surgery.

Surgery among jaundiced patients avoiding PBD needs to be organized quickly and therefore there is a significant reduction in the time from presentation to surgery (58, 76). Often patients do not require tissue diagnosis for this strategy thus patients who can proceed directly to surgery may also avoid the need for preoperative tissue diagnosis, required for patients to receive NAT. Thus, if surgery for BRPC can be planned to provide a realistic chance for an R0 resection, defined above as being limited to those with BR-venous disease only, there are multiple potential benefits of an upfront surgery pathway. Furthermore, the resection rate is clearly higher when surgery is performed within a week or two after diagnosis rather than when there are delays (11–13, 77).

The patient experience may therefore be improved within a direct to surgery approach due to avoidance of PBD, for some patients the avoidance of need for tissue diagnosis and rapid progression through a treatment pathway. One criticism of upfront surgery is that surgery is associated with significant risks. However, it can be seen that risks of pancreatic fistula, the major determinant of morbidity and mortality after pancreatoduodenectomy are lower following surgery for PDAC than other indications (78) and, furthermore, centralization and modern treatment algorithms are associated with much reduced rates of perioperative mortality (1). Among those patients with resectable PDAC the survival of patients with upfront surgery who do not receive adjuvant therapy appears similar to those receiving NAT who did not undergo surgery (19) i.e., for those patients that ultimately do not complete their treatment pathway there is no apparent survival advantage with either NAT or upfront surgery. There is no quality of life data from randomized trials to directly compare the patient experience. However, from studies of FOLFIRINOX in the palliative setting there is a significant detrimental effect upon patient experience (79).

There may be concerns over the safety of venous resection in the setting of jaundice however this has been studied and amongst 100 patients undergoing venous resection, 36 underwent surgery in the presence of jaundice. There was no significant difference in complications, length of stay or 90 days mortality between the cohorts of patients stratified by associated venous resection (Pande et al., unpublished).

## Future Developments

There is clearly a strong drive for NAT. Precision medicine offers the promise of individualized patient care and strategies such as the PrecisionPanc platform will provide ways for clinicians and researchers to undertake studies to advance our understanding of tumor biology and selection of patients to different therapies as a result. In this regard it is essential to support these studies and there will be an increasing need to collect preoperative tissue via EUS. Well-conducted clinical trials are needed that are designed to include “real world” patients and not groups of highly selected patients otherwise results may be misleading and ungeneralizable. Study design may need to be novel to include adaptive design to allow therapies to evolve over the course of the study. Finally collaboration is key to ensure timely patient recruitment.

## Conclusions

Treatment of BRPC is under intense scrutiny with much interest placed upon the role of NAT. NAT offers undoubted improvements in R0 rates and survival among those patients completing surgery (80). However, treatment and selection bias mean that these outcomes must be considered in the wider context of the full patient pathway and ability of patients to receive these treatments. On an ITT basis there is presently no clear advantage with a neoadjuvant approach for those with BRPC and isolated venous involvement. This is an important note, the studies of patients with BRPC must differentiate those with and without arterial involvement. Upfront surgery of those with arterial disease is highly unlikely to achieve an adequate chance of R0 or survival benefit whilst NAT for those with BRPC and arterial disease offer a chance for an R0 resection and acceptable duration of survival.

Upfront surgery for BRPC and venous disease can offer advantages in terms of avoiding preoperative biliary drainage whilst preoperative biliary drainage is mandated among those who undergo NAT. These advantages are yet to be considered in the setting of BRPC and venous disease and must be considered within any future trial of upfront surgery vs. NAT. Furthermore, the nature of chemotherapy delivered in published cohort studies of neoadjuvant or adjuvant settings are not the same; this treatment bias favors those patients receiving NAT. However, improvements in surgical outcomes need to be appreciated and the duration and toxicity of NAT must be considered. The aggressive nature of PDAC commands both a rapid and radical approach though well-designed randomized trials are needed to truly determine survival advantages of these approaches. *Primum non nocere*.

## Author Contributions

All authors listed have made a substantial, direct and intellectual contribution to the work, and approved it for publication.

### Conflict of Interest

The authors declare that the research was conducted in the absence of any commercial or financial relationships that could be construed as a potential conflict of interest.
