# Supplementary material for: Determining Optimal Routes to Surgery for Borderline Resectable Venous Pancreatic Cancer—Where Is the Least Harm and Most Benefit?
Source: Front Oncol. 2019 Oct 17;9:1060. doi: 10.3389/fonc.2019.01060 (PMC6811510; doi:10.3389/fonc.2019.01060)
Supplement: Supplementary file 1 [file Image_1.pdf]

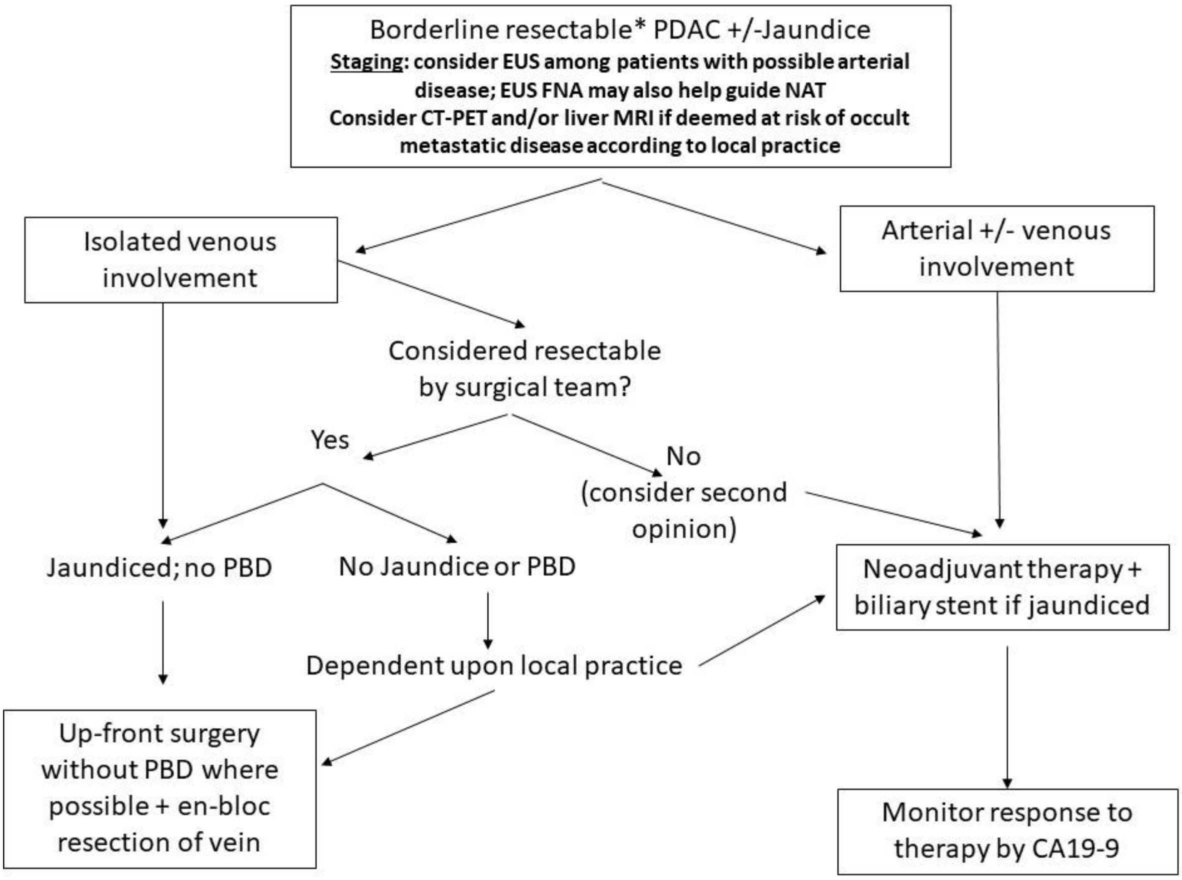

Figure 1: Algorithm for borderline resectable PDAC. *Borderline resectable PDAC defined as:

<u>NCCN/ISGPS</u>

•Borderline: venous involvement of the SMV/PV with distortion or narrowing of the vein or occlusion of the vein with suitable vessel proximal and distal allowing for safe resection and replacement tumour contact >180 degrees or contact <180 degrees, with contour irregularity or thrombosis and suitable for recon; SMA: solid contact <180 degrees or short segment contact

•Locally advanced: Unreconstructable SMV and/or PV due to tumour involvement or occlusion OR Contact with most proximal draining jejunal branch into SMV.

<u>AHPBA/SSAT/SSO/NCCN</u>

•SMV/PV abutment/impingement/encasement/short segment occlusion.

•SMA abutment HA abutment or short segment encasement CA uninvolved

<u>MD Anderson</u>

•SMV occlusion SMA abutment HA abutment or short segment encasement CA abutment

<u>Alliance Vein</u>

•Tumour-vessel interface ≥ 180° of vessel wall circumference,
and/or reconstructable occlusion SMA: Tumour-vessel interface < 180° of vessel wall circumference
